# Evidence from the Kaduna State Health Accounts on the pattern of sub-national health spending in Nigeria, 2016

**DOI:** 10.1136/bmjgh-2019-001953

**Published:** 2020-05-05

**Authors:** Chukwuemeka Emmanuel Azubuike, Yewande Kofoworola Ogundeji, Nuha Butawa, Nneka Orji, Paul Dogo, Kelechi Ohiri

**Affiliations:** 1 Health Strategy and Delivery Foundation, Abuja, Federal Capital Territory, Nigeria; 2 Kaduna State Ministry of Health and Human services, Kaduna, Nigeria; 3 Federal Ministry of Health, Abuja, Nigeria

**Keywords:** health insurance, health economics, health systems, public health

## Abstract

Health accounts provide accurate estimates of health expenditure, which are important for effective resource allocation and planning in the health sector. In Nigeria, four rounds of health accounts have been conducted at the national level. However, the national estimates do not necessarily reflect realities at the subnational level and may only provide limited information for decision making at that level. This study highlights the pattern of health spending in Kaduna State from the 2016 Health Accounts, with a view to providing more reliable evidence for decision making in the state.

Health accounts expenditure surveys were administered to government, donors, non-governmental organizations (NGOs), private health insurance organisations and employers in the health sector for the reference year 2016. Household health expenditure was derived from a household survey administered across a representative sample of 1024 households selected from six local government areas across the three senatorial districts in the state. We estimated disease expenditure by deploying a health provider survey across a sample of 100 health facilities. Analysis was conducted using Microsoft Excel, Stata and the Health Accounts Production Tool.

Findings show that current health expenditure (CHE) accounted for only 7% of the total health expenditure in 2016. Out-of-pocket spending among households was about 81% of CHE, compared with a national average of 71.5% of CHE between 2010 and 2014. The health expenditure findings highlight several policy imperatives for the Kaduna State Health System. Primary among these is the heavy dependence on out-of-pocket financing for health, which has negative implications on vulnerable households. A shift to pooled prepaid mechanisms would reduce the financial burden on the most vulnerable households in Kaduna State. In addition, considering the government’s current contribution to health expenditure, there is a strong need for increased government prioritisation of the Kaduna State health sector.

**Significance of the study**

**What is already known?**

- Out-of- pocket payments constitute the major mechanism of financing healthcare in Nigeria.

**What are the new findings?**

- Evidence on health spending is now publicly available in Kaduna State, providing insight on the pattern of sub-national health spending in Nigeria.
- The burden of out-of- pocket payments is slightly higher in Kaduna compared with the national average for Nigeria.
- A high proportion of health expenditure was on pharmaceuticals, mostly purchased from private patent medicine vendors.

**What do the new findings imply?**

- There is an urgent need to shift towards pooled prepaid mechanisms for funding healthcare in Kaduna, and other states in Nigeria.
- Findings from this study have the potential to generate new learnings for other states in Nigeria and the Federal government of Nigeria.

## Introduction

As health systems aspire towards universal health coverage (UHC), healthcare decision makers are constantly faced with certain financing questions, such as ‘How much is spent on health?’ and ‘Who pays for what?’.^1^ Health accounts present a useful approach to answering these critical health financing questions by analysing the health system from an expenditure perspective.^1^

Health accounts, produced and used routinely in many high-income countries for decision making, provide a systematic description of financial flows related to the consumption of healthcare goods and services.^1^ In low-income and middle-income countries, health accounts are increasingly conducted to generate evidence on the state of health financing.^1^ In addition, evidence from the National Health Accounts (NHA) in many countries has led to several policy reforms on health insurance, increased prioritisation of health, reprioritisation of public spending and earmarked taxes.

In Nigeria, four rounds of health accounts have been successfully conducted at the national level between 1998 and 2016. These revealed low government spending and high out-of-pocket health expenditure disproportionately borne by households.^2^ The findings from the 2010–2014 NHA were important inputs into the policy dialogues that ultimately resulted in the signing into law of the National Health Act 2014. This Act provides a legal framework for the regulation, development and management of Nigeria’s health system.

In spite of this, evidence suggests that national level health accounts findings do not necessarily reflect the realities at the subnational level, especially for countries like Nigeria with a decentralised government and health system structure. Thus, while NHA findings may be reliably used to inform resource allocation and health planning at the national level, health accounts studies at the subnational level are also required to inform decisions and planning at state levels. This rationale has spurred a few of the states in Nigeria such as Anambra, Bauchi, Sokoto and Kaduna to conduct their first System of Health Accounts to aid evidence-based decision making.^3^

This paper profiles Kaduna State (see box 1) and presents information on health spending in the state, the sources of funding and a description of funds that flow through the health system based on the health accounts framework, with a view to providing more reliable evidence for decision making in the Kaduna Health system.

Box 1 **An overview**

Kaduna State is one of 36 states in Nigeria; it is in the north-western part of the country and is the third most populous state with a projected population of 8.3 million in 2016. The state is predominantly rural; on average, 50% of rural household members are engaged in agriculture compared to 15% in urban households, while an estimated 22% of the labour force is unemployed. Poverty rate was estimated at 56.5%% in 2018 based on the multidimensional poverty index. The state is composed of 23 local government areas which are categorised into three geosenatorial zones.

The health system in Kaduna is decentralised from the national level with administrative oversight provided by the State Ministry of Health. Private participation also exists from donors, non-governmental organisations (NGOs) and health insurance organisations. These organisations play an important role in providing and maintaining access to healthcare across the over 1200 public and private facilities in the state. The state government funds the provision of health services in state-owned health facilities, while federal health institutions domiciled in the state receive funding from the federal government. Donors and NGOs are also involved in providing health services through faith-based health facilities and donor-supported public health facilities in the state. Health indices from the National Demographic and Health surveys (NDHS), 2013 revealed that Kaduna State lagged behind similar economically active states such as Lagos, Enugu and Rivers in the access to basic maternal, neonatal and child health services.^7^

This low level of performance in the health sector spurred the government in 2015 to undertake a data-driven primary healthcare system diagnostic to uncover the root causes of underperformance in the state. The diagnostic revealed, among other things, weak budgeting, poor financial management and lack of transparency around resource flows in the health system, and provided a basis to conduct several demand-side analytical studies in the state.

This study presents a profile of the health financing system for the year 2016 in the state using the System of Health Accounts (SHA) 2011 framework to answer specific policy questions:

- How are resources mobilised and managed for the health system?
- Who pays for health and how much is paid?
- How much do development partners contribute financially to the health system?
- Which health providers receive health expenditure?
- How much is spent on primary healthcare in Kaduna State?

## Methods

### Overview and approach

The Kaduna State health accounts process adhered to the internationally recognised framework for health accounting—the System of Health Accounts 2011,^1^ as well as the implementation manual for health accounts in Nigeria.^4^ For the purposes of health accounts in Nigeria, healthcare expenditure is defined as that which ‘encompasses all expenditures for activities whose primary purpose is to restore, improve and maintain health during a defined period’.^4^ This definition is applicable regardless of the type of the institution or entity providing or paying for the health activity.^4^

The System of Health Accounts framework delineates health expenditures based on the core accounting framework into financing schemes, functions and providers as well as capital formations. Around the core framework, an extended accounting classification further delineates expenditures according to factors of provision, revenues of financing schemes, financing agents and diseases^1^ (see table 1).

**Table 1:** A summary of the System of Health Accounts dimensions

Code	SHA dimension	Description
FSRI	Revenue institutions	Institutional units providing revenues to financing schemes
FS	Revenues of financing schemes	How revenues (ie, funds) are mobilised by financing schemes (type of revenues)
HF	Financing schemes	Financing arrangements through which people obtain health services
FA	Financing agents	Institutional units/organisations who operate the healthcare financing schemes (ie, manage the healthcare funds)
HP	Healthcare providers	Organisations and actors who deliver healthcare goods and services
HC	Healthcare functions	The type of need healthcare transactions aim to satisfy OR the kind of objective pursued
DIS	Disease class	The disease which an activity/expenditure line links to
FP	Factors of provision	Inputs needed to produce healthcare goods and services
HK	Capital account (separate mapping)	Total value of the fixed assets that health providers have acquired during the accounting period (less the value of the disposals of assets)

Within the context of the Kaduna State health accounts, and based on the type of revenues, public funds consist of funds from federal government revenue for health entities operational in Kaduna State, also state government revenues for state institutions; as well as donor funds channelled through government budget mechanisms. The private funds are composed of funds from households, employers, private health insurance firms as well as donor funds spent through donors or non-governmental organisations (NGOs).

Previous health accounts methodologies aggregated total current and capital formations (expenditure) as total health spending. However, the SHA 2011 framework provides a separate treatment of capital formation to avoid the ambiguity regarding links between current health spending and capital health spending.^1^

### Data collection

We used data from primary and secondary surveys administered to institutions, households and healthcare facilities in the state including private enterprises.

#### Health Accounts Production Tool Survey and supporting surveys

To estimate health expenditure from government institutions, donors, private health insurance organisations, employers and NGOs, we identified a data universe of 85 organisations. Standardised health accounts survey questionnaires that were generated from the Health Accounts Production Tool (HAPT) were then administered to the 85 organisations in the data universe. The data were collected by trained enumerators with supervision from the State Ministry of Health, Kaduna State Bureau of Statistics and the implementing partner supporting the SHA study.

##### Government expenditure data

Data were collected from the 31 public sector institutions providing care and receiving health funds. The survey administration to government organisations was supplemented with secondary data collection from the government audited accounts and other relevant government financial statements. Expenditures by the public sector organisations consisted of those incurred through disbursement to various government entities, expenditures in own operated health facilities, and so on.

##### Donor and NGO expenditure data

Donor and NGO surveys were administered to the 8 donors and 21 NGOs identified by the health partners coordination forum in the state to capture their contributions for health. The data sources captured sources of funds across multilateral, bilateral and private donors as well as the recipients of the funds and what services were purchased on behalf of consumers.^4^

##### Employers (enterprises) expenditure data

Enterprises in the state within the context of the health accounts are private organisations with more than 10 staff members whose primary activity is not health, but which provide some form of health coverage for their employees.^1^ We selected all eight enterprises in the state that met the SHA definition from a reference list of enterprises. This reference list was developed using a list of private enterprises from the Bureau of Statistics supplemented by expert judgement of the state health financing technical working group. The employer survey captured employers’ expenditures with regards to payment of employees’ health insurance premiums, external health provider contracting and provision of healthcare services in own healthcare facilities for employees and dependents.^4^

##### Private health insurance expenditures (health maintenance organisations)

Lastly, surveys were administered to the 17 health maintenance organisations (HMOs) accredited by the national health insurance scheme in the state in 2016. The data captured health spending on beneficiaries enrolled under health insurance plans.

#### Household survey

To capture household contribution to health spending in the state, as well as estimate current levels of out-of-pocket spending for health in Kaduna State, a household out-of-pocket expenditure survey was conducted. A representative sample of 1024 households in six local government areas across the three distinct geosenatorial zones was canvassed using a three-stage sampling method. The sampling frame consisted of 21 792 enumeration areas across the state and constituted the primary sampling unit. This sampling frame is consistent with that used for the 2009/2010 Harmonised National Living Standards Survey; and the 2013 National Demographic and Health Survey.^5^

The first stage of sampling consisted of a stratified random sampling of 6 LGAs out of the 23 LGAs in the state to select 1 urban and 1 rural LGA in each geosenatorial zone. The geosenatorial zones are distinct from each other in language, household size, income, religion, living standards. In the second stage, probability proportionate to size sampling was conducted to select 17 enumeration areas per LGA, and in the third stage, 10 households were selected in each enumeration area. The survey assessment tool captured relevant information such as: household characteristics and consumption, health service utilisation, inpatient/outpatient expenditures, access to health insurance and willingness to pay for health insurance.

### Ethical approval for the household survey

All the study personnel were trained on relevant aspects of good ethical standards relevant to the survey. Study participants were given adequate information about the study to enable them to take an informed decision about participating in the study. This was done verbally and without pressure or inducement from the study personnel. The right to abstain from participation in the study at any time, even during the study, without any negative consequences, and the confidential nature of responses were emphasised. The interviews took place within the household compound and respondents were given the opportunity to suggest any conducive place where confidentiality is ensured, and distractions minimised. Participants’ names were not recorded anywhere in the final data sets; instead, we used a unique identification number.

#### Health facility survey

To derive disease-related expenditures and proportions, health facility data were collected across selected facilities in the state. A representative sample of 100 public health facilities was selected across primary, secondary and tertiary tiers of care using a three-stage sampling approach which was stratified by senatorial zone, urbanity and level of care. Data extracted included routinely available data from health facility registers and the health management information system, such as: monthly summary information for all months of 2016, and outpatient and inpatient attendance for 4 months in 2016 (January, February and August, September). The time period was selected to control for variation in disease burden between the dry (November to March) and rainy (April to October) seasons. To estimate intensity of resource use (as a proxy for cost), user fees incurred in providing health services was captured during the survey.

### Patient and public involvement statement

Patients and members of the public were not used in the design, conduct, reporting and dissemination of this research. Patient utilisation data were collected from health facilities and analysed for the health provider survey; however, individual patients cannot be identified from the reported data.

### Data analysis

Data analysis was conducted using Microsoft excel, STATA and the HAPT. Data from donors, NGOs, enterprises and private health insurance firms were first cleaned and then imported to the HAPT. A response rate of 61% was achieved across organisations that received the HAPT survey as shown in table 2.

**Table 2:** Distribution of survey respondents across institutions and their respective response rates

Domain	Surveyed	Responded	% Response
Government	31	24	77.4%
Donor	8	6	75%
Non-governmental organisation (NGO)	21	15	71.4%
Employer	8	6	75%
Health maintenance organisation	17	1	5.8%
**Total**	**85**	**51**	**61%**

Due to the general reluctance of HMOs in Kaduna to release expenditure data for the particular year, a low response rate of 5.8% was achieved across all 17 HMOs in the state. The household and health provider surveys achieved response rates of 96% and 72%, respectively.

Government expenditure for the health sector was extracted on an excel sheet. These data were then translated to a government code book to align the data with government economic codes and render the data amenable to the HAPT. Government expenditure line items across the various institutional entities were distributed and mapped to the SHA categories based on the description of the spending.

Donor and NGO data were also subjected to critical assessment. In instances where donors reported disbursements to NGOs, and NGOs reported receiving funds from the same donor, the donor spending was excluded as the NGO expenditure (financing agent) is regarded as closer to the actual expenditure compared with donor-reported expenditure. The currency of analysis was the Nigerian naira.

The SHA methodology allows for weighting in the instance of non-response to surveys. To account for entities that either were not surveyed or did not return a completed survey, weights were applied to adjust the survey responses on the HAPT. Considering the low response rate among HMOs, we estimated total private health insurance expenditure by triangulating data from the NHA, published literature and the Kaduna State Health Accounts Study. We compared the reported private insurance claims expenditure of 5,300,000 naira and claims rate of 75% from the only respondent HMO, to the average claims rate in Nigeria from two published studies which reviewed claims data across three HMOs between 2010 and 2011, and found consistency with the figures.^6 7^

Household survey data were cleaned and analysed using STATA statistical software. Descriptive characteristics of the surveyed populations were computed using proportions and means. Cross-tabulation of data was done to assess inter-relationships between variables—notably assessing health expenditures, healthcare utilisation, willingness to pay across asset wealth quartiles, expenditure groupings, sex and age. Average annual per capita OOP was calculated and multiplied by total population of the state to derive total OOP. This was then disaggregated into health account dimensions of providers, functions and factors of provision using splits/proportions derived from the household survey.

To derive disease-specific expenditure proportions using the health facility data, first the diagnoses reported in the provider survey were translated to International Classification of Diseases format. This was conducted independently by two medical professionals on the team. Results were compared, and conflicts regarding categories or diagnosis were resolved. Second, the disease categories were translated to health accounts disease codes. Third, a cost analysis was conducted using the user fees incurred in providing services across facilities. The average user fee of each standardised disease was estimated by two independent analysts on the team. Fourth, we estimated total visits per year per disease category leveraging utilisation data from the provider survey, as well as vaccines and HIV/AIDS data from DHIS2. Lastly, we derived disease-related spending and expenditure proportions by multiplying visits per year by average user fee incurred in providing treatment.

## Results

### Aggregate and current health expenditure

In older health accounts methodologies, aggregate health expenditure was the sum of current health expenditure (CHE) and capital expenditures on health (capital formations).^1^ Within the context of the current SHA 2011, current expenditure refers to final consumption of goods and services by all entities, while capital formation refers to final consumption of capital goods by health providers. SHA 2011 thus separates both capital and current expenditures as they constitute different expenditures and refer to different timings of consumption. Hence, computations in this paper are based on total current expenditure on health in Kaduna in 2016. All currency exchange rates reflect those as at commencement of the study in February 2017.

At 181,481,953 989 billion naira ($595 million), the total current expenditure on health accounted for 98% of aggregate health spending in the state. This current expenditure figure also translates to 7% of the 2.5 trillion naira gross domestic product (GDP) of Kaduna State in 2016, and represents a per capita health spending of 21,865 naira ($71).

### Total CHE by financing sources

**Figure 1:**
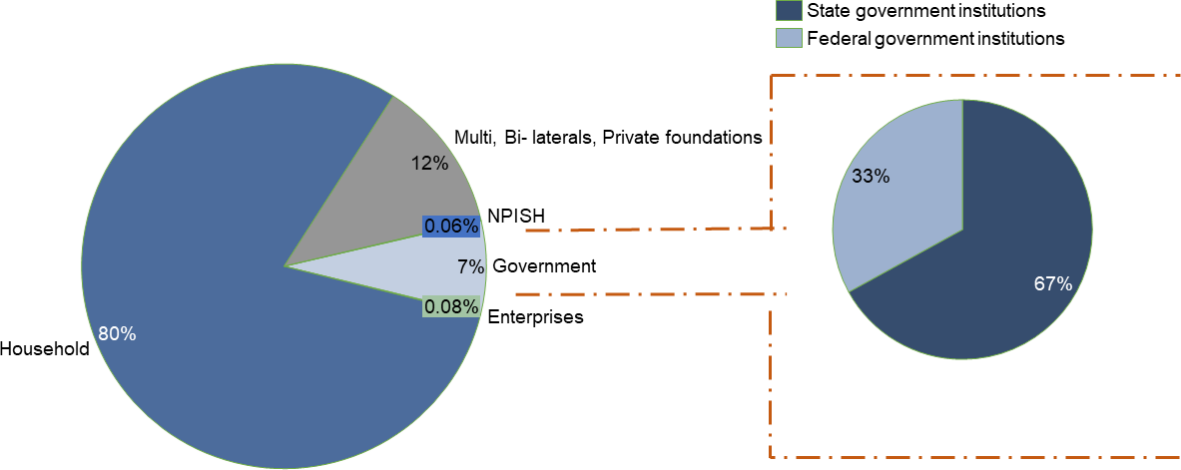
Disaggregation of total expenditure on health in Kaduna State—the total current expenditure = 181,481,953,989 Naira; and further disaggregation of the proportion of current government spending by federal and state government institutions.

The Kaduna State health sector is broadly financed from private sources, mainly household out-of-pocket payments. As depicted in figure 1, the estimated household OOP spending was about 145 billion naira ($475 million), representing about 80% of CHE. Public financing of healthcare accounted for 13 billion naira ($42 million) constituting about 7% of the total CHE, with 67% attributed to state government institutions and 33% to federal government institutions. Donors’ and employers’ contributions constituted 22 billion naira ($72 million) and 148 million naira ($485,000) accounting for 12% and <0.1% of CHE, respectively. Non profit institutions serving households (NPISH) also constituted <0.1% of CHE.

### Total CHE by financing schemes

**Figure 2:**
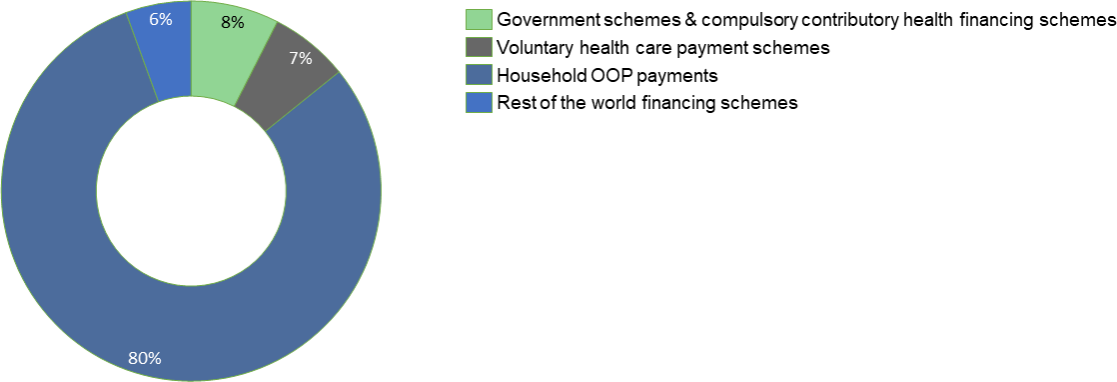
Disaggregation of current health expenditure by financing schemes.

As presented in figure 2, out-of-pocket payment schemes remain the dominant means of paying for healthcare in Kaduna State, constituting over 80% of total CHE by financing mechanisms. Government schemes and compulsory contributory healthcare financing schemes constituted only 8% of the total CHE. Voluntary healthcare payment schemes through voluntary health insurance, employer-based insurance schemes and enterprise financing schemes constitute 7% of CHE. The rest of the world financing schemes through philanthropy/international NGO schemes, and foreign development schemes constituted 6% of CHE in Kaduna State.

### Total CHE by health providers

**Figure 3:**
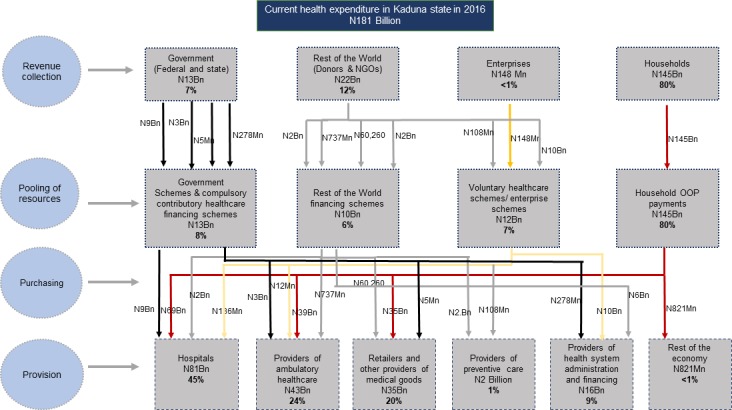
Flow of money in the Kaduna State health system in 2016.

**Figure 4:**
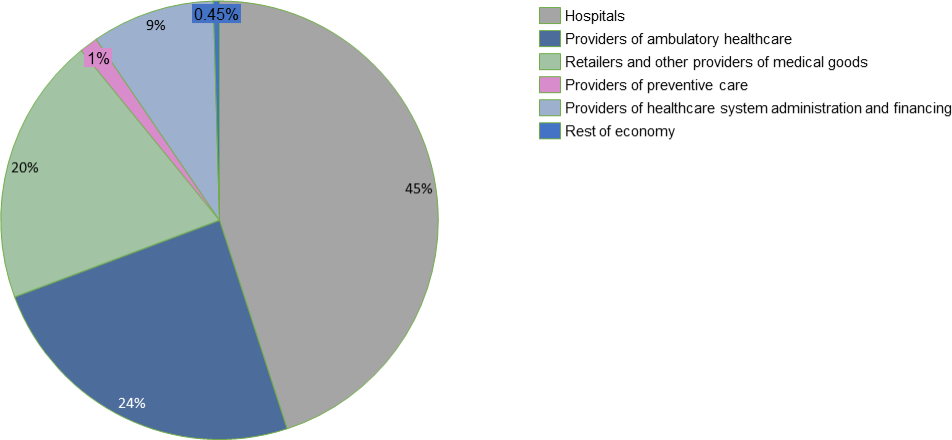
Disaggregation of current health expenditure by providers of healthcare.

Figure 3 shows the flow of healthcare naira in the Kaduna State health system from financing sources through schemes, to providers of healthcare. Figure 4 represents the distribution of CHE by healthcare providers. As depicted in both figures, hospital care accounted for 45% of CHE, providers of ambulatory care services (Primary health care facilities) constituted 24% of CHE, retailers and other providers of medical goods (pharmacies and chemists) accounted for 20% of CHE.

Health system administration and financing which entails regulatory activities provided by agencies such as the State Ministry of Health and State Primary Healthcare Development Agency contributed 9% of CHE. Preventive care activities through collective preventive programmes, campaigns, and so on, in family planning centres and youth friendly centres accounted for only 1% of CHE, while provision of healthcare through community health workers, and through households as providers of care was valued at <1% of CHE.

Figure 3 also shows that 88% of the spending in hospitals was paid for through household OOP payments, while 11% of hospital health spending was paid for through government schemes. Almost 100% of health spending by retailers and other providers of medical goods was financed through household OOP payments. A comparison of health expenditure by types of healthcare facilities revealed that general hospitals account for 53% of CHE while primary healthcare centres and tertiary hospitals account for 35% and 12%, respectively.

### Total CHE by healthcare functions and factors of healthcare provision

Curative care was the dominant function on which health expenditures were incurred, accounting for 90% of current health spending in Kaduna State, while preventive care accounted for 7% of CHE. A further disaggregation of curative care by type showed that over 65% of curative healthcare expenditure was incurred at outpatient departments, and 28% at inpatient departments in hospitals in Kaduna State.

With regards to factors used in providing healthcare, about 23% of total current expenditure could not be assigned to specific factors of healthcare provision due to poor reporting of the expenditure data by the respondents. Of the remaining 77% (139 billion naira/$455 million) assignable to specific factors of provision, 88% was spent on materials and services used, 10% on salaries and wages and 2% on consumption of fixed capital. Further disaggregation of material and services showed that expenditure on pharmaceuticals was the major driver of spending on materials and services in the state.

### Total current health expenditure by diseases

**Figure 5:**
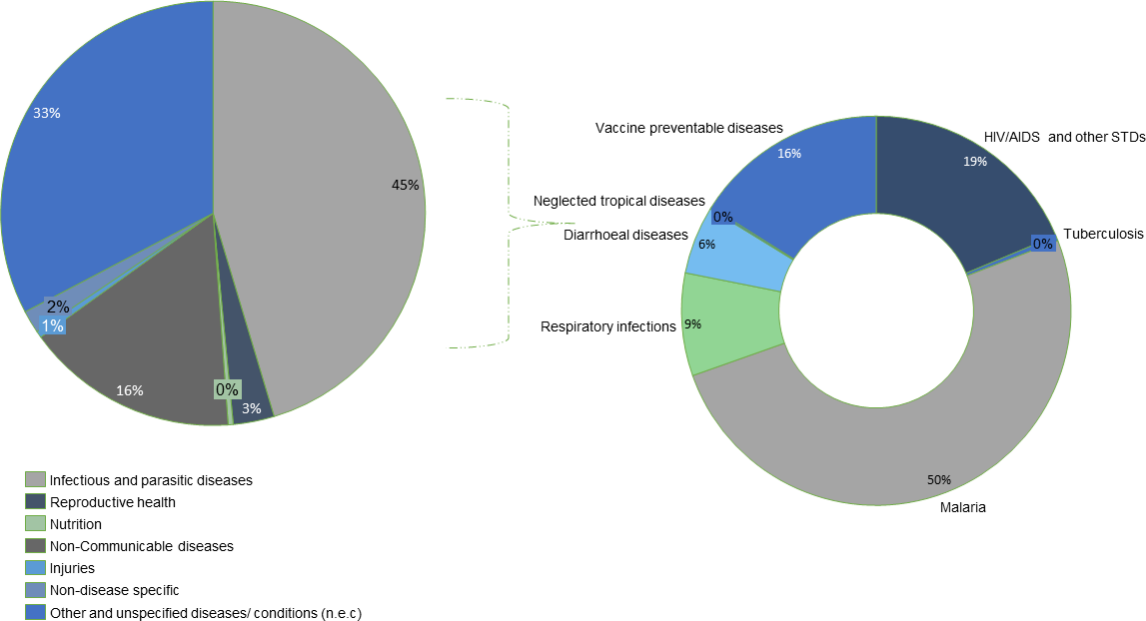
Disaggregation of current health expenditure by diseases and further disaggregation of infectious disease expenditure by subclasses of infectious disease.

Figure 5 shows that about 45% of CHE (82 billion naira/$268 million) was spent on infectious and parasitic diseases, 16% of CHE (29 billion naira/$95 million) was spent on non-communicable diseases and 3% of CHE (5 billion naira/$16 million) on reproductive health. Thirty-three per cent of CHE was not assignable to specific diseases also due to poor categorisation of the expenditure data by the respondents. Disaggregating infectious disease expenditure by types shows that malaria, HIV/AIDS with sexually transmitted diseases, and vaccine preventable diseases were the key drivers, accounting for 50%, 19% and 16% of infectious disease spending, respectively. See online supplementary table 1 for spending proportion by disease.

10.1136/bmjgh-2019-001953.supp1 Supplementary data

## Discussion

This paper provides a profile of the Kaduna health financing system from an expenditure perspective. The findings show that at 7% of GDP, the total health expenditure to GDP ratio in Kaduna State was higher than the national average of 4%, and international target reference of 5%. On a per capita basis, findings also show that the government expenditure of 1,617 naira (US $5) was lower than the recommended US $84 per capita by the High level task force on innovative financing for health systems (HTLF) to deliver a basic package of primary health services in low-income countries.^8^

The evidence of a high health spending proportion relative to GDP should be interpreted with caution. Although it appears nominally positive, it does not necessarily imply sufficiency or adequacy, and could even be misleading, considering that the total health expenditure was mostly borne out of pocket by households in Kaduna State which already face a high poverty incidence^9^ and are therefore prone to catastrophic expenditure. This relationship between poverty and catastrophic health spending is also bolstered by evidence from China and several countries in sub-Saharan Africa which show an inverse relationship between economic status and catastrophic health expenditure.^10 11^ This presents an imperative for the Kaduna State government to effectively target vulnerable groups in the design of financial protection mechanisms in the state.

Government spending on health constituted only 7.5% of the total government budget.^12^ When compared with the Abuja Declaration which recommended at least 15% of the total budget for health,^13^ there might be an argument for increased prioritisation of the health sector to ensure sustainability of healthcare financing in the state. However, given the variation and ongoing debate on thresholds of what countries should spend on health, this finding should be interpreted with caution. Notwithstanding, it is important that increased public spending on health translates into financial protection for Kaduna citizens towards UHC. Increasing public spending is dependent on the state government’s revenue which may be prone to volatility as a resultof an over-reliance on statutory allocations from the federal government which already depend on revenues from dwindling oil prices. Increased government prioritisation of health in Kaduna would thus require an understanding of the fiscal space for health in the state from the existing, latent sources and from new sources.

Private financing of care mainly through household out-of-pocket payments constituted 80% of total health expenditure in Kaduna state. This has implications for catastrophic health spending especially for the poor and vulnerable in the State. This finding also resonates with those in many sub-Saharan African countries where household OOP has been found to be the dominant means of health financing, accounting for between 72% and 99% of all private sources, often leading to catastrophic spending.^14^

High OOP payments for health have been found to have catastrophic and impoverishing effects on household living standards.^15^ A recent household OOP expenditure survey in Kaduna State found that about 57% of households incurred catastrophic expenditure.^13^ This implies that about 6 in every 10 households have health expenditures that exceed 10% of the household income with a higher distribution among poorer households.^16^ In the context of a high poverty incidence (56.5%),^8^ the poor and vulnerable in Kaduna are exposed to huge financial risk as a result of health outlays. It is important that households in Kaduna experience a shift in the burden of health financing through a more sustainable, pooled, prepaid mechanism. Effective implementation of the new Kaduna State contributory health insurance scheme is a step in the right direction as demonstrated in many low-income and middle-income countries including Namibia, Costa Rica and Peru.^17^

Furthermore, in Kaduna State, households were the main agents in the health financing space, and this is unsurprisingly due to the high share of household spending in the total health expenditure in the health sector. From a health accounts perspective, the overall functioning of a health financing system entails transactions that are executed by the financing agents (purchasers) according to the rules of the financing schemes.^1^ Effective health financing systems should have institutional agents which mobilise and manage funds to meet the current and future health needs of the population.^1^ With households as the dominant financing agents, participating in transactions based on their immediate decisions to use health services, ability to pay at the time of use and with no pooling mechanism, meeting the future health needs of the citizens in the state may be difficult. Thus, it is important that health financing reform in the state emphasises a greater role of government in financing care especially through well designed prepaid risk pooling mechanisms where premium sfor the poor and vulnerable are subsidised.

The NHA 2014 conducted by the Federal Ministry of Health, showed that donor funds constituted about 13% of total health expenditure.^2^ This finding is marginally higher than our study finding which revealed that external funding from donors constituted about 12% of the total health expenditure, but is lower than the average share of external spending on health of 20% across 20 sub-Saharan African countries according to the 2019 global health spending report^18^. Relative to the 7% government expenditure on health in Kaduna, donor expenditure at 12% is indicative of some level of reliance on donors. An over-reliance on external donor funding has drawbacks which include a lack of predictability, selective focus on vertical programming, large number of actors to be coordinated, and short-term horizons for most commitments.^19^ Given macroeconomic realities in many states in Nigeria, health system issues and donor interests in those contexts, donor financing may be necessary in the short to medium term to improve and sustain health system goals. However, in the long term, such external financing should only be supplementary to the government’s active prioritisation and should gradually transition to more sustainable and predictable funding approaches.

Preventive care accounts for only 7% of health expenditure while curative care accounts for 90%, and this indicates that spending for the delivery of healthcare in Kaduna State might be inefficient relative to disease burden. In addition, our study revealed low expenditure in primary health facilities of 24% compared to hospital spending of about 44%. This suggests that citizens may not be satisfied with service provision in primary health care facilities and therefore circumvent PHCs for more expensive hospital-based care. This impacts service structure efficiency, and may have negative implications, considering that Nigeria has committed to strengthening primary healthcare as a means of achieving UHC. Households also accounted for 100% of expenditure made to retailers and other providers of medical goods, that is, pharmacies and private patent medical vendors, many of which are easily accessible to households but are not properly regulated and monitored in the health system^20^. It is therefore imperative that effective policies are implemented to regulate and monitorthe activities of these private patent medical providers in the state to assure qualityand safety of procured drugs.

This study has some limitations. First, due to data collection issues experienced among some respondents in the health system, alternative approaches within the context of the system of health accounts methodology were employed to adjust the data for non-responses. Second, due to the low response rate of 5.8% across HMOs, we extrapolatd the claims rate across all HMOs in Kaduna state and upweighted the claims expenditure to account for the HMOs that did not respond. This approach is top-down as it does not factor in variations in enrolment figures across HMOs in Kaduna State. In addition, using a single point claims rate of 75% may lead to wrong estimation of expenditures, especially for HMOs with a very low or high claims rate.

Third, estimating intensity of resource use for the provider survey was quite difficult in the absence of published costing studies on service provision in health facilities, or time motion studies to estimate staff time. Health facility staff were reluctant to report on the actual cost of health services, due to concerns that the data would be used for compliance and quality assurance purposes. Hence, we used collected data on user fees as a proxy for cost and intensity of resource use

## Conclusion

The results of this study provide useful evidence for health financing reform in Kaduna. Previously unavailable evidence on the profile of health expenditure in the state, expenditure on providers of health services, primary healthcare spending, donor contribution to health and disease-related expenditure are now available for use in reform planning in the Kaduna State health sector.

Our study shows the urgency with which political actors and policy makers in Kaduna State need to increase government prioritisation for health in the state and design robust financial risk protection mechanisms to reduce the high out-of-pocket expenditure for health among citizens. Evidence suggests that increased allocation of public funds to the health sector leads to a decrease in OOP health expenditure as well as catastrophic health spending. In the absence of a state supported financial risk protection mechanism, high OOP spending implies that many citizens likely face catastrophic and impoverishment consequences of seeking healthcare.

The study also highlights a need to strengthen primary healthcare financing in the state. A reallocation of resources towards primary healthcare will signal that the state government is ready to spend more efficiently and sustainably in providing access to affordable and quality health services to citizens. The imperative for primary healthcare reform and mainstreaming has been given renewed political commitment with the 2018 Astana declaration for primary healthcare,^21^ to which Nigeria is a signatory. The Kaduna health system will benefit from better financing for PHC as a means of achieving its UHC aspirations.

Key recommendations based on the findings of this study highlight important policy imperatives for the Kaduna health sector and these are summarised in table 3.

**Table 3:** Key findings and potential policy implications of the Kaduna State health accounts 2016

Key findings	Policy implications
A very high proportion of health spending is borne by households, which exposes Kaduna citizens to catastrophic expenditure.	For Kaduna to achieve the recommended benchmark of 30% OOP as a percentage of THE, it is critical that the newly signed into law contributory health insurance scheme is well designed, successfully implemented and financially sustainable. The scheme will ensure that Kaduna households are protected from the financial shock of seeking and paying for healthcare.
Government contribution to health spending is relatively low.	Government spending onhealth can be improved by (1) Increasing allocation of resources towardshealth. (2) Adequate cash backing of health budgets by the state government. The State Ministry ofHealth can facilitate this by demonstrating value for money in healthcarespending and actively engage the Ministry of Finance and the Commission ofBudget and Planning in ensuring cash backing and release of budgeted funds.
Preventive care accounts for a negligible proportion of current health spending while parasitic diseases including malaria, HIV/AIDS and vaccine preventable diseases are the major expenditure drivers.	A shift from curative to preventive care especially at the PHC level may reduce healthcare costs for the system while improving overall health of citizens in the long run. Kaduna State and Nigeria have a lot to gain from innovative financing for preventive health services. The Astana declaration which Nigeria is a signatory to, provides a renewed political commitment for policy makers and political actors to prioritise disease prevention and health promotion services as a means of improving health and strengthening PHC.
Less than 25% of government HE was spent in PHCs. This is quite low given that PHCs cater to more than 60% of the population of the state.	Kaduna State can reprioritise and reallocate resources towards PHCs in the future through successful implementation of the service delivery plan and cash backing for operational expenses at PHCs. In addition, a successful roll-out and implementation of the basic healthcare provision fund (an earmarked fund aimed at improved government financing of both supply and demand sides of basic health services) at both federal and state levels will contribute to improving allocation of funds toPHCs.

Some of these recommendations are currently being implemented in Kaduna State. For example, Kaduna State is currently piloting a contributory health insurance scheme for citizens in the state, with significant subsidies for maternal and child health services for pregnant women and children under 5 years. This would improve financial protection especially for the vulnerable populations. In addition, a fiscal space analysis of the Kaduna health sector in 2018 revealed several potential sources of revenue for health in the state. These sources include earmarked taxes, premiums from the state contributory health scheme, efficiency gains from improved financial management and improved budget implementation. Further exploration of some of these sources would increase government expenditure in the health sector.
