# Supplementary material for: Evidence from the Kaduna State Health Accounts on the pattern of sub-national health spending in Nigeria, 2016
Source: BMJ Glob Health. 2020 May 5;5(5):e001953. doi: 10.1136/bmjgh-2019-001953 (PMC7228467; doi:10.1136/bmjgh-2019-001953)
Supplement: Supplementary data [file bmjgh-2019-001953supp001.pdf]

Disease	Disease SHA code	Spending proportion
Malaria	DIS.1.3	0.16
Typhoid	DIS.1.nec	0.20
Undiagnosed fever	DIS.nec	0.08
Pregnancy	DIS. 2.1	0.04
Other illness	DIS.nec	0.33
Respiratory infection	DIS 1.4	0.04
Diarrhea	DIS 1.5	0.01
Chronic Conditions	DIS 4	0.07
Accident/injections	DIS 5	0.03
Skin conditions	DIS.nec	0.01
HIV/AIDS/STD	DIS 1.1	0.01
Family planning	DIS 2.3	0.01
Preventive services	DIS 6	0.01
